# Determination of genes and microRNAs involved in the resistance to fludarabine *in vivo *in chronic lymphocytic leukemia

**DOI:** 10.1186/1476-4598-9-115

**Published:** 2010-05-20

**Authors:** Etienne Moussay, Valérie Palissot, Laurent Vallar, Hélène A Poirel, Thomas Wenner, Victoria El Khoury, Nasséra Aouali, Kris Van Moer, Bernadette Leners, François Bernardin, Arnaud Muller, Pascale Cornillet-Lefebvre, Alain Delmer, Caroline Duhem, Fernand Ries, Eric van Dyck, Guy Berchem

**Affiliations:** 1 Laboratory of Experimental Hemato-Oncology, CRP-Santé, Luxembourg, Luxembourg; 2 Microarray Center, CRP-Santé, Luxembourg, Luxembourg; 3 Human Molecular Genetics, Cliniques universitaires saint-Luc/de Duve Institute/Université Catholique de Louvain, Brussels, Belgium; 4 Société pour la Recherche contre le Cancer et les Maladies du Sang, Luxembourg, Luxembourg; 5 Hematology Department, University Hospital, Reims, France; 6 Centre Hospitalier de Luxembourg, Luxembourg, Luxembourg

## Abstract

**Background:**

Chronic lymphocytic leukemia (CLL) cells are often affected by genomic aberrations targeting key regulatory genes. Although fludarabine is the standard first line therapy to treat CLL, only few data are available about the resistance of B cells to this purine nucleoside analog *in vivo*. Here we sought to increase our understanding of fludarabine action and describe the mechanisms leading to resistance *in vivo*. We performed an analysis of genomic aberrations, gene expression profiles, and microRNAs expression in CLL blood B lymphocytes isolated during the course of patients' treatment with fludarabine.

**Results:**

In sensitive patients, the differentially expressed genes we identified were mainly involved in p53 signaling, DNA damage response, cell cycle and cell death. In resistant patients, uncommon genomic abnormalities were observed and the resistance toward fludarabine could be characterized based on the expression profiles of genes implicated in lymphocyte proliferation, DNA repair, and cell growth and survival. Of particular interest in some patients was the amplification of *MYC *(8q) observed both at the gene and transcript levels, together with alterations of myc-transcriptional targets, including genes and miRNAs involved in the regulation of cell cycle and proliferation. Differential expression of the sulfatase *SULF2 *and of miR-29a, -181a, and -221 was also observed between resistant and sensitive patients before treatment. These observations were further confirmed on a validation cohort of CLL patients treated with fludarabine *in vitro*.

**Conclusion:**

In the present study we identified genes and miRNAs that may predict clinical resistance of CLL to fludarabine, and describe an interesting oncogenic mechanism in CLL patients resistant to fludarabine by which the complete *MYC*-specific regulatory network was altered (DNA and RNA levels, and transcriptional targets). These results should prove useful for understanding and overcoming refractoriness to fludarabine and also for predicting the clinical outcome of CLL patients before or early during their treatment.

## Background

Chronic lymphocytic leukemia (CLL), the most common hematologic malignancy in Western countries, is characterized by the accumulation in the blood of monoclonal CD5^+ ^CD19^+ ^CD23^+ ^B lymphocytes mainly arrested in G_0_/G_1 _phase of the cell cycle. Various cytogenetic abnormalities are often found in CLL B cells. Importantly, a few conserved aberrations are consistently observed in association with rapid disease progression and short survival times [1]. These aberrations affect chromosome 12 (trisomy), 13q14 (microRNA-15a (*miR-15a*)/*miR-16-1*), 17p13 (*TP53*) and 11q22 (*ATM*). CLL B cells fail to fulfill their immunological role and are not able to achieve either their final differentiation or programmed cell death.

Due to their potential to kill non-dividing cells [2], purine nucleoside analogs such as fludarabine (9-b-D-arabinofuranosyl-2-fluoroadenine) are often used as first-line therapy to prolong the progression-free survival of CLL patients. In addition to the use of fludarabine as a single chemotherapeutic agent, new attractive therapies combining fludarabine with chimeric monoclonal antibodies were recently successfully introduced.

The mechanisms underlying the toxic effects of fludarabine have been studied *in vitro*. In proliferating cells, fludarabine requires DNA synthesis for cytotoxicity and induces apoptosis in a cell cycle-dependent manner by inhibiting several enzymes involved in DNA metabolism [3]. In quiescent human lymphocytes, fludarabine incorporation inhibits DNA synthesis during DNA repair and thus induces irreversible damage triggering disruption of mitochondrial integrity and apoptosis [4,5]. The p53 signaling pathway, which provides a major control mechanism to regulate cell cycle arrest and apoptosis in response to DNA damage, is also involved in fludarabine-mediated cytotoxicity. Post-translational modifications of p53 regulate its activity and determine cell fate by modulating the class of target genes inducing G_1 _or G_2 _arrest, DNA repair and/or apoptosis depending on the extent and severity of damages [6]. Killing of resting lymphocytes was previously reported to act by p53-independent and -dependent manners *in vitro *and *in vivo *[2]. In addition to p53-related aspects, the resistance to cancer treatment in CLL has been connected to biochemical alterations targeting membrane transporters, deoxycytidine kinase, and cytoplasmic 5'-nucleotidase cN-II activities, and more recently to changes in the levels of miR-34a [7], a miRNA belonging to the class of small non-coding RNA molecules mediating post-transcriptional gene silencing. MicroRNAs regulation is currently the subject of intense research, and differential expression of miRNAs has been reported in B cells of healthy donors and CLL patients [7-10].

Previous *in vitro *studies of fludarabine were based on the treatment of cells in culture and did not take in consideration parameters like pharmacokinetics and cell microenvironment which represent critical factors for the outcome of the treatment *in vivo*. Although fludarabine has been known for twenty years to have considerable activity against CLL [11], its mode of action at the molecular level *in vivo *is only poorly described. Thus, half the cases of resistance that manifest after several rounds of fludarabine treatment [12] cannot be explained by p53 defects [13]. In this study, we have addressed the response of CLL B cells to fludarabine *in vivo*. We have investigated the mechanisms involved in the resistance to fludarabine and identified molecular markers predictive of poor clinical responses. Rare genomic aberrations were detected that target genes playing prominent roles in the regulation of important cellular mechanisms. We report on a set of genes and miRNAs that could predict the clinical outcome of CLL patients and refine prognosis before chemotherapeutic treatment. Finally, we discuss the perspectives offered by our findings for improving CLL therapeutic strategies, overcoming resistance in CLL patients and enhancing the overall survival of previously treated patients with impaired prognosis.

## Methods

### Patients and samples

This research was approved by the Comité National d'Ethique de Recherche (Luxembourg, N°200509/05) and participants gave written informed consent in accordance with the Declaration of Helsinki. CLL was diagnosed according to standard clinical and laboratory criteria. Patients received fludarabine (25 mg/m^2 ^for 5 days per cycle, Fludara^®^, Bayer Healthcare Pharmaceuticals) during their therapy in the departments of hematology/oncology (Centre Hospitalier du Luxembourg and University Hospital of Reims). Clinical details, previous treatments and genomic characteristics of patients (N = 18, CLL-1 to -18) are summarized in Additional file 1. CLL patients showing an important decrease in lymphocyte count after treatment and not requiring further therapy were classified as sensitive (called S) to fludarabine. Other patients were classified as refractory to the treatment (called R) as they showed only a moderate decrease of their lymphocyte count after treatment, relapsed very quickly, and required further therapies. Patient CLL-3R died because of CLL complications two months after the beginning of the treatment. The treatment of patient CLL-2S was stopped because of a cytomegalovirus re-activation. In addition, a validation cohort of CLL patients (N = 21, CLL-19 to -39) was used to confirm the prognostic role of genes and miRNAs on the evaluation of the resistance to fludarabine *in vitro*.

Peripheral blood samples were collected from patients before their treatment (T0) for patients CLL-1 to -18, and after 1 to 9 days of fludarabine treatment *in vivo *(T1, T2, and T9, for patients CLL-1 to -6 only), and from CLL patients recruited for the *in vitro *validation (CLL-19 to -39). The isolation of mononuclear cells was performed from whole blood by density gradient centrifugation using the Lympho isolation medium (MP Biomedicals, France). From the collected cells, the proportion of B cells was always greater than 90%. Otherwise, a negative selection was performed to eliminate T and NK cells and monocytes/macrophages with help of the Dynabeads^®^CD2 and CD14 (Dynal Biotech, Norway), respectively.

### Cytotoxicity assay

Cell viability was measured by using the tetrazolium salt-based Cell Counting Kit-8 assay (Dojindo Molecular Technologies, Inc., Eke, Belgium). Cells from patients CLL-19 to -39 were washed twice in PBS and cultured at a density of 1.5 × 10^6^/ml (100 μl per well in 96-well plates) in RPMI-1640 supplemented with 10% of fetal calf serum. Cells were treated for 48 h with a range of fludarabine (Fludara^®^, Bayer Healthcare) concentrations (0-10 μM), including clinically achievable concentrations (1.5 - 4.5 μM, [14]). CCK-8 reagent was then added and plates were incubated before measuring the absorbance at 450 nm.

### DNA isolation, array-based CGH, cytogenetics, and FISH analyses

All genetic analyses were performed on sample T0 prior to treatment. Comparative Genomic Hybridization (CGH) array analysis was performed to identify genomic aberrations in B cells of patients CLL-1 to -6 only. Briefly, DNA of selected B cells was isolated using the QIAamp DNA Blood Mini Kit (QIAGEN) following the instructions of the manufacturer. After enzymatic digestion (AluI and RsaI), 2 μg of patient DNA and control Human Genomic DNA (Promega) were coupled with the Genomic DNA Labeling Kit PLUS (Agilent, France) to cyanine 5 and 3, respectively, and then hybridized (40 h at 65°C) on Agilent 60-mer 244 K Human Genome CGH Microarray following protocols of the manufacturer. Images were analyzed with Feature Extraction^® ^software (Agilent) and data analysis was performed with CGH-Analytics^® ^(Agilent) using the Aberration Detection Methods ADM1 and ADM2.

Conventional cytogenetic analyses were available for patients CLL-1 to -18. Fluorescence in situ hybridization (FISH) analysis was performed using the LSI ATM-2/CEP11, LSI p53/CEP17, LSI S13S319 with CEP 12, LSI IGH/BCL2, LSI IGH/CCND1 and LSI PML/RARA fluorescent probes (Vysis/Abbott, Belgium) according to standard protocols.

### RNA isolation

The procedure followed was similar for cells from patients treated *in vivo *and cells incubated *in vitro*. The B cells were centrifuged at 350 *g *for 5 min at 20°C and the pellet was lysed in Trizol^® ^(Invitrogen) following the instructions of the manufacturer with slight modifications. Briefly, after addition of chloroform and vigorous shaking, the samples were transferred into 1.5 ml-Phase Lock Gel Heavy tubes (Eppendorf) and centrifuged for 10 min (12,000 *g *at 4°C). The aqueous phase was recovered and 5 μg of RNase-free glycogen were added as carrier. Total RNA was precipitated with isopropyl alcohol overnight at -20°C, centrifuged at 12,000 *g *for 10 min at 4°C, and washed once with 75% ethanol. After treatment with DNase (DNA-*free*™, Ambion), RNA quantity and quality were evaluated with the NanoDrop^® ^ND-1000 spectrophotometer and the Agilent 2100 bioanalyzer, respectively.

### TP53 and Immunoglobulin heavy-chain sequence analysis

We analyzed DNA samples from CLL patients by PCR and fluorescent sequencing to determine *TP53 *mutational status (exon 2-10) using Big Dye Terminator kit and ABI 3130 sequencer (Applied Biosystems). The primer sequences are available upon request.

The B lymphocyte Ig gene rearrangement and mutational status was determined by IgVH-specific RT-PCR and sequencing [15]. Complementary DNA (cDNA) was obtained by reverse transcription of 2 μg RNA by using the Reverse Transcriptase Core kit with random nonamers (Eurogentec, Belgium). Fifteen microliters of cDNA were amplified by PCR using a mixture of primers [15] specific for VH1-VH6 sequences together with either a 3' primer specific for the JH consensus region or a 3' primer specific for the constant region of the IgM locus. Amplification products were gel-extracted and sequenced. Nucleotide sequences were compared to the international ImMunoGeneTics information system^® ^(IMGT/V-QUEST) and to the NCBI Ig BLAST databases. The homology of the sequence with the closest germline counterpart was assessed and sequences with 98% or greater homology to germline-encoded VH gene were categorized as unmutated.

### Complementary DNA microarrays

Microarray experiments were performed with samples from CLL patients sensitive (CLL-1, -2, -4, -5) and resistant (CLL-3 and -6) to fludarabine. As the treatment of patients can be assimilated to a time course experiment, the commonly used two-channel microarray experiment loop design was chosen [16]. Each sample was hybridized to two different samples in two different dye orientations (T0 *vs *T1, T1 *vs *T2, T2 *vs *T9, T9 *vs *T0) in quadruplicates. Briefly, 1 μg of total RNA from B cells of patients CLL-1 to -6 only was amplified and labeled with the Amino Allyl MessageAmp™ II aRNA Amplification Kit (Ambion, UK). The coupling reaction was performed following the protocol of the manufacturer using Alexa Fluor^® ^Reactive Dye Decapacks (AF555 and 647, Molecular Probes). Alexa Fluor^®^-aRNA were hybridized for 16 h on cDNA microarrays (Operon human version2 oligonucleotide library, University Medical Center Utrecht, The Netherlands). Microarrays were scanned at 10-μm resolution with two laser channels (532 and 635 nm) using a GenePix^® ^4000B scanner and GenePix^® ^Pro 6.0 (Molecular Devices Corporation, CA) at variable photomultiplier tube (PMT) gains (< 1% saturated spots).

Microarray data were deposited in the EMBL-EBI ArrayExpress public repository (Accession number E-MTAB-70). The image analysis and spot selection were performed with MAIA 2.7 (Institut Curie, France) as previously described [17]. Acuity^® ^4.0 (Molecular Devices, CA) was used for data processing (dye-swap, Lowess normalization), warehousing and visualization. A few microarrays were excluded from analysis based on the results of Pearson cross-correlation coefficient (data not shown). Data were subjected to statistical examination with the SAM add-on for Microsoft Excel (Significance Analysis of Microarrays, Stanford, CA; using centered median, 100 permutations). Missing values were imputed using the *k*-nearest neighbor method (*k *= 10). Delta values were adjusted to obtain gene lists with a false discovery rate (FDR) ≤ 1% for one-class analysis and ≤ 5% for two-class analysis. Genes identified by SAM two-class analysis as regulated in CLL B cells after 24 h of treatment with fludarabine *in vivo *were classified by enriched gene sets with the Gene Set Enrichment Analysis algorithm (GSEA 2.05, database Dec 2009; Broad Institute, MA). Differentially regulated genes were clustered as molecular interaction networks with Ingenuity Pathway Analysis (IPA 8.0, database Nov 2009; Ingenuity Systems, CA).

### Quantitative RT-PCR

For validation of microarray data, specific primers were purchased from Eurogentec (primer sequences available upon request). Five nanograms of cDNA were used in a 25 μl PCR reaction mixture containing 12.5 μl SYBR^® ^Green PCR MasterMix, and 300 nM of each primer. PCR amplifications were performed on ABI 7300 Real-Time PCR System (40 cycles at 95°C - 15 sec and at 60°C - 60 sec). Values were normalized according to previous recommendations [18] to both *28S rRNA *and *FLOT2 *by using the average Ct value of housekeeping genes and were processed by using the Ct value obtained before treatment as calibrator (2^-Δ ΔCt ^calculation method). MicroRNAs to be tested were chosen as i) they were reported as regulated in CLL ii) they modulate genes involved in apoptosis and cell cycle iii) they are known transcriptional targets of Myc. The detection of miRNAs was performed by TaqMan^®^-based qPCR by using 50 ng of total RNA as starting material. The TaqMan miRNA Reverse Transcription and TaqMan 2X Universal PCR Master Mix, No AmpErase UNG together with specific TaqMan miRNA Assays (all Applied Biosystems) were used for RT and PCR reactions (35 cycles at 95°C - 15 sec and at 60°C - 60 sec). All miRNA expression values were normalized to both *18S rRNA *and *FLOT2 *(2^-ΔΔCt ^calculation method). ZAP-70 and LPL quantification were performed as described before [19].

## Results

### Common genomic abnormalities observed before fludarabine treatment

Eighteen CLL patients were given one to six fludarabine cycles (25 mg/m2 per day for 5 days) and classified as sensitive or resistant to the treatment based on changes in lymphocyte counts, relapse, and requirement for further therapy. We first analyzed common genomic abnormalities in samples collected prior to treatment. Results obtained by array-based CGH analysis and targeted genes are presented in Table 1 and Additional file 1. Classical abnormalities affecting 13q14, 11q22, chr 12, and 17p13 were detected. Sequencing of *TP53 *gene indicated the presence of a V274G point mutation in the exon 8 of B cells from patient CLL-5S. In patient CLL-3R, a 2nt-deletion in exon 7 induced the presence of a stop at codon 239 (truncated protein). In patient CLL-6R, although one allele was deleted, the second one did not present any mutation (Additional file 1). Interestingly, among the ten patients clinically resistant to fludarabine, five (50%) presented with wild-type *TP53 *gene, underlying the importance of factors other than p53 in the mechanisms leading to resistance to this drug. In addition, no influence of ZAP-70 expression, IgVH status or deletions affecting 11q or 13q on the response to treatment could be identified. In summary, no difference was observed between patients in regards to their sensitivity or resistance to the treatment when taking in consideration classic clinical parameters and history of previous treatments.

**Table 1:** Genomic aberrations and targeted genes observed in CLL patients resistant to fludarabine.

Genomic aberrations	Type of aberration	Targeted gene(s)
idic(3q)	Deletion 3p	*XPC, RAD18, PCAF*
	Gain 3q	*BCL6, FAIM, p63*
7q21, 7q36 ^1^	Gain	*MDR family genes*
idic(8q)	Deletion 8p	*TNFRSF10 *family genes
	Gain 8q	*MYC*
9p24p21 ^2^	Deletion	*JAK2, p16INK4*, *CDKN2B, SMARCA2*
11q23	Deletion	*ATM*
12p13.31p12	Deletion	*p27*
13q14.2q14.3	Deletion	*DLEU *genes, *Rb*, miR-15a, miR-16-1
15q15q26	Gain	*MGA*, *PML, BLM, cyclin B2*, and *53BP1*
idic(17q)	Deletion 17p	*p53, MNT*
	Gain 17q	*STAT3, SPOP, RAD51L3, RARA, RDM1*

^1 ^These aberrations were observed only in patient CLL-6R

^2 ^This aberration was observed only in patient CLL-3R

### Genetic complexity of resistant CLL B cells before fludarabine treatment

Although the B cells from most patients displayed genomic imbalances (Additional file 1), conventional cytogenetics and CGH-arrays indicated that the extent and complexity of these chromosomal aberrations were higher in B cells from the resistant patients, as illustrated by the losses, gains, and dicentric isochromosomes (idic(17)(p12) and idic(8)(p12) observed in patients CLL-3R and CLL-6R. Thus, a gain on 15q15.1q26.3, where the genes *MGA*, *PML*, *BLM*, *cyclin B2*, and *53BP1 *are located (Table 1), was detected in both patients by CGH and confirmed by FISH analysis (Additional files 2 and 3). We also identified a deletion of 9p24.3p13.1 targeting *JAK2*, the tumor suppressors *CDKN2A *and *CDKN2B *and *SMARCA2 *(*Brm*) in patient CLL-3R. Finally, deletions on 6q14.1q14.3, 14q24.2q24.3 and 14q32.11q32.12 and alterations on chromosome 7 (*MDR *genes) rarely observed in CLL were also detected in B cells of patients CLL-3R and -6R, respectively.

### Gene expression profile in CLL B cells in response to fludarabine treatment in vivo

**Figure 1:**
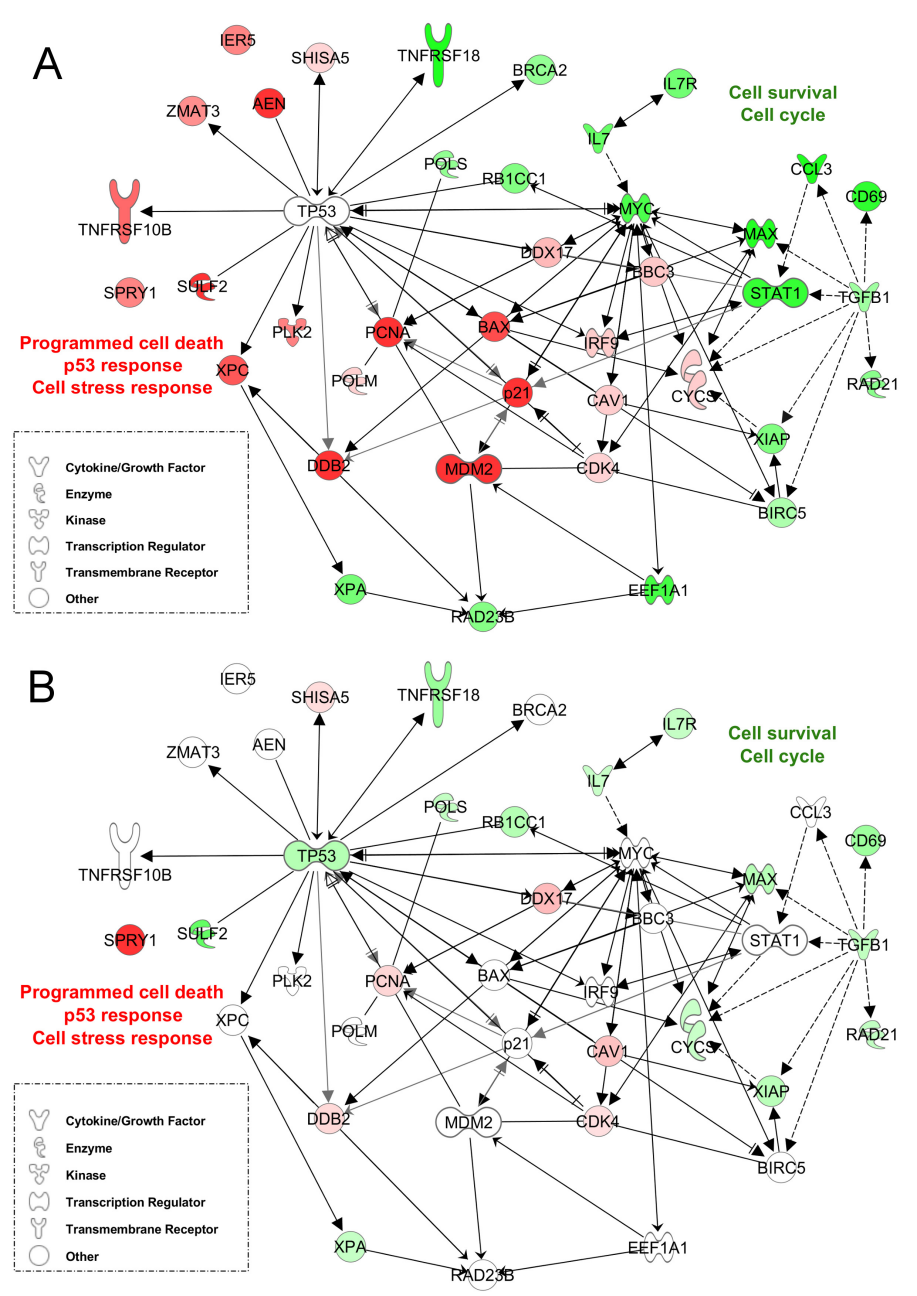
**Molecular interaction network of fludarabine-regulated genes in cells of sensitive CLL patients *in vivo***. A group of genes highly regulated by fludarabine in sensitive patients (following a 24h exposure *in vivo*) was clustered in a network around the transcription regulators *TP53 *and *MYC *using the Ingenuity Pathway Analysis software (IPA 8.0, database Nov 2009). ***A***, Gene expression values obtained from sensitive patients were overlaid on the network. ***B***. Gene expression values obtained from resistant patients were overlaid on the network.

To gain more knowledge about the mechanisms underlying the cellular response to fludarabine, we next examined changes in gene expression profiles induced by fludarabine *in vivo*. To this end, we identified genes whose expression was altered by fludarabine in B cells from six patients given a fludarabine cycle (5 days). Samples were collected prior to treatment as well as 1, 2 and 9 days following the first administration. A one-class SAM analysis was run to specify differentially expressed genes in each patient and time transition in response to the treatment. The four sensitive patients responded similarly to the treatment at all time points. In addition, the responses after 24/48h of treatment were very similar. Fludarabine-regulated genes (FDR ≤ 1%) together with their biological function and fold changes are listed in the Additional file 4. Pathway analysis indicated that most of genes identified in this study as regulated were involved in programmed cell death, cell cycle, p53 signaling, cell stress response, response to DNA damage, and cell-to cell signaling and interaction (p < 0.05, data not shown), confirming previous studies [4,5,15,20]. Therefore we focused our investigations on these biological pathways. We noted that most of regulated genes were p53 or Myc transcriptional targets. Therefore a molecular interaction network centered on both transcription factors was created with the most regulated genes after 24h of fludarabine treatment *in vivo *(Fig. 1A). Interestingly, the p53 response was highly induced (dark red) by fludarabine as indicated by the strong up-regulation of *p21*, *PLK2*, *cyclin G1*, *DDB2*, and *XPC*, consistent with the notion that p53 signaling provides an important pathway leading to cell cycle arrest and cell death in response to fludarabine. Regulators targeting p53 for degradation and important cell growth regulators were also over-expressed after 24/48h of treatment. On the other hand, a strong down-regulation of several genes was induced by the treatment in all sensitive patients (dark green). Among these were genes regulated by the *MYC *and *STAT1 *transcription factors involved in cell survival (*XIAP *and *BIRC5/*survivin), cell growth and proliferation (*IL7, IL7R*), *and *DNA repair and replication. Lymphoid markers, chemokines, cytokines, and their receptors were also down-regulated.

A more detailed list of regulated genes and functions can be found in the Additional file 4.

After 9 days of treatment, a different molecular response was observed; most p53-target genes were now down-regulated, whereas other genes involved in the control of growth and apoptosis were still up-regulated (data not shown).

### Gene expression profile in resistant CLL B cells in vivo

#### SAM one-class analysis

**Figure 2:**
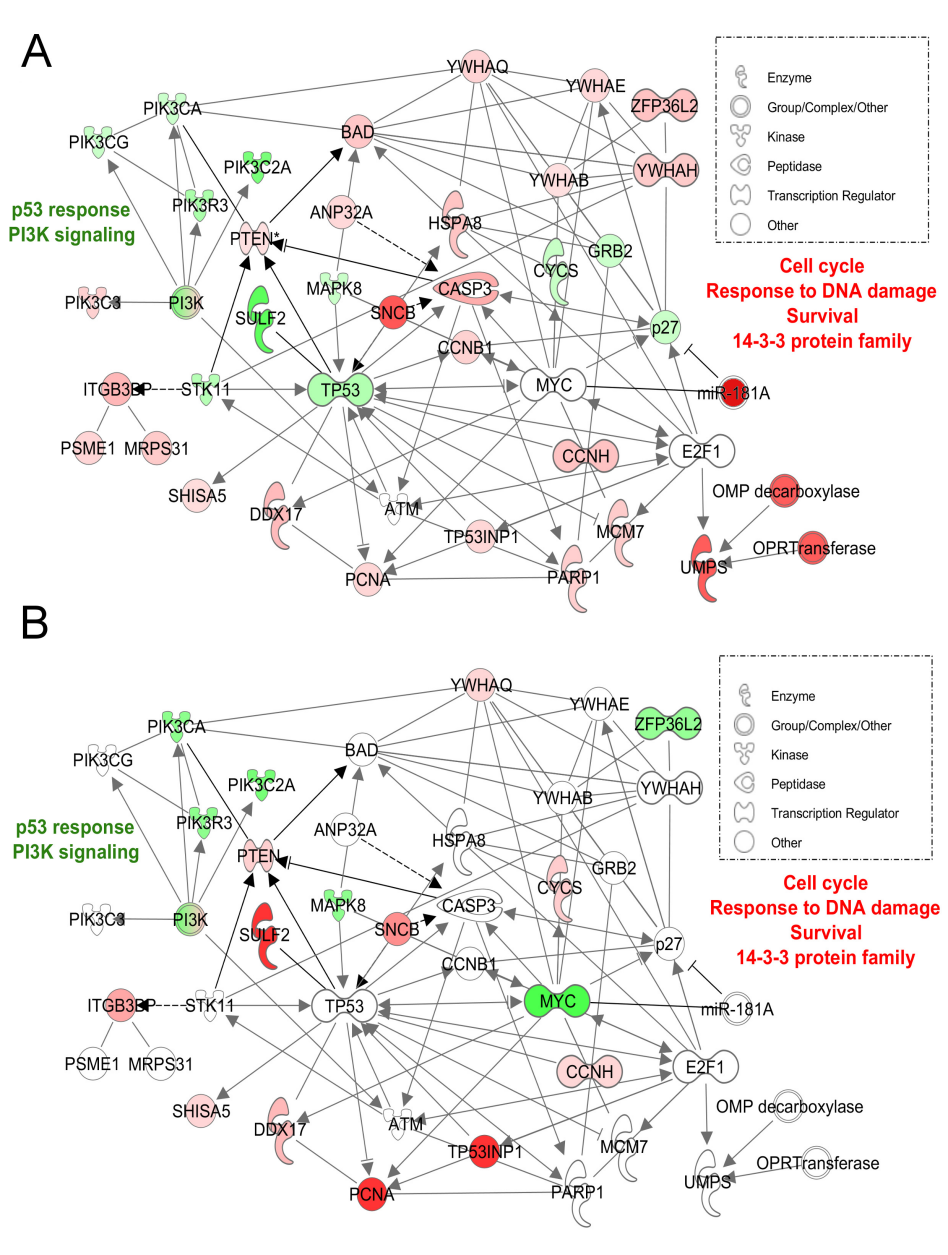
**Molecular interaction network of fludarabine-regulated genes in cells of resistant CLL patients *in vivo***. A group of genes highly regulated by fludarabine in resistant patients (following a 24h exposure *in vivo*) was clustered in a network around the transcription regulators *TP53 *and *MYC *using the Ingenuity Pathway Analysis software (IPA 8.0, database Nov 2009). ***A***. Gene expression values obtained from resistant patients were overlaid on the network. ***B***. Gene expression values obtained from sensitive patients were overlaid on the network.

An overlay of gene expression data obtained in cells of resistant patients was performed on the molecular interaction network built with gene expression of sensitive patients. We observed strikingly different molecular responses as few p53-target genes were regulated in cells of resistant patients (Fig. 1B). In contrast, other genes were regulated by MYC and p53 in B cells of resistant CLL patients (Fig. 2A). The conserved families of PI3K and 14-3-3 proteins were highly regulated in B cells of resistant patients together with cell cycle and cell death/survival regulators. Genes significantly regulated in resistant patients were also involved in nucleoside and nucleotide metabolism (*UMPS*), the response to DNA damage (*PARP1*) and replication (*MCM7*), in contrast to what observed in cells of sensitive patients (Fig. 2B). A more detailed list of regulated genes and functions can be found in the Additional file 5.

#### SAM two-class analysis

**Figure 3:**
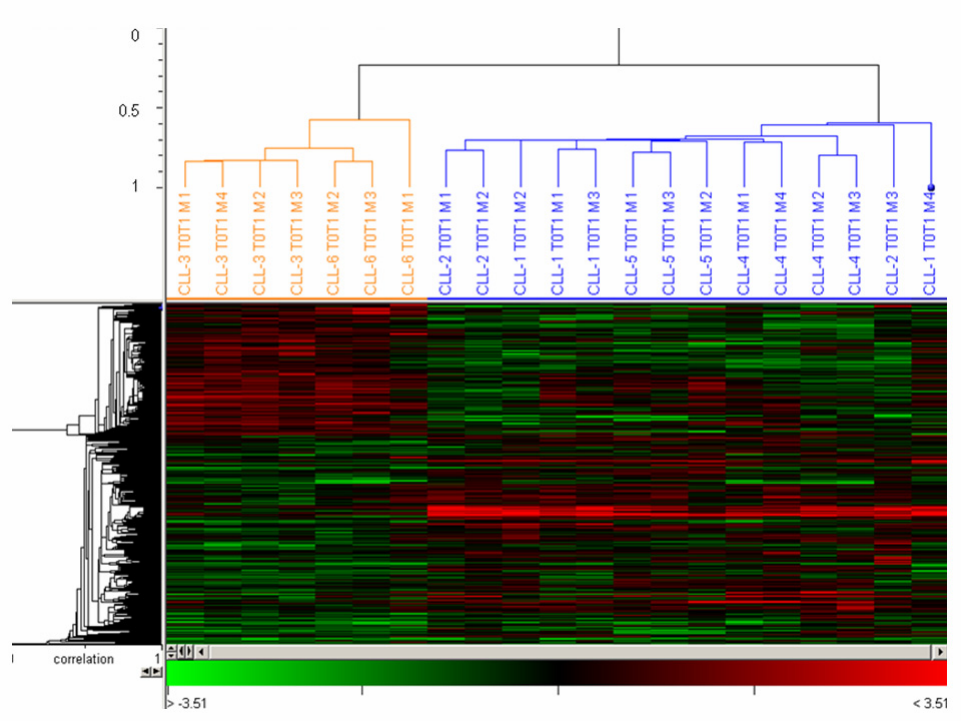
**Clusters of differentially expressed genes in CLL B-cells following treatment with fludarabine *in vivo***. A set of 532 genes were differentially expressed (DE) between CLL B cells of sensitive and resistant patients after 24 hours of treatment. Statistical analysis was performed by SAM 2-class analysis and significant DE genes were clustered using Acuity 4.0 (Pearson hierarchical clustering). Microarray replicates (M1 to M4) from sensitive and resistant patients are depicted in blue and orange, respectively.

A direct comparison of the responses elicited by fludarabine in CLL B cells from sensitive and resistant patients was performed by SAM two-class analysis followed by unsupervised Pearson hierarchical clustering, revealing dramatic differences in the patterns of gene expression induced by the drug. As shown in Fig. 3, a set of 537 genes was significantly differentially regulated between cells of resistant and sensitive patients after 24h of fludarabine treatment. The genes identified by SAM analysis can be found in the Additional file 6. Among these genes, 207 were over-expressed in resistant while repressed in cells of sensitive patients. Conversely, 330 genes were induced in sensitive while repressed in cells of resistant patients. These genes were classified by gene sets and biological functions with the GSEA algorithm. The genes up-regulated in B cells of sensitive patients were mainly involved in p53 signaling, DNA damage response, DNA repair, cell death, and cell cycle (Table 2). These functions were appreciably different from those represented in resistant patients in whom the majority of genes were implicated in the control of lymphocyte proliferation, cell growth and survival, DNA replication and repair, and functions of mature lymphocytes. Gene sets also contained responses mediated by *MYC, EGR2*, and *APC *transcription factors.

**Table 2:** Gene set enrichment analysis (GSEA) of CLL B cells from fludarabine-sensitive and -resistant patients

Function/Pathway	Gene sets enriched in sensitive patients	Size	NES	NOM p-val
p53 response	STRESS_ARSENIC_SPECIFIC_UP	6	1.76	0.002
	P53PATHWAY	6	1.61	0.004
	HSA04115_P53_SIGNALING_PATHWAY	13	1.62	0.006
	P53GENES_ALL	9	1.53	0.021
	P53_BRCA1_UP	5	1.50	0.030
	STRESS_P53_SPECIFIC_UP	5	1.44	0.031
	KANNAN_P53_UP	9	1.51	0.032
DNA damage response	DNA_DAMAGE_SIGNALING	13	1.69	0.008
	UVC_TTD-XPCS_COMMON_DN	8	1.69	0.014
	CIS_XPC_DN	10	1.73	0.017
DNA repair, cell cycle, apoptosis	BLEO_HUMAN_LYMPH_HIGH_4HRS_UP	9	1.59	0.028
BAFF/APRIL receptor	MOREAUX_TACI_HI_VS_LOW_UP	8	1.45	0.023
hTERT-regulated genes	SMITH_HTERT_DN	5	1.57	0.028
	ROTH_HTERT_DIFF	7	1.48	0.049
NF-KB signaling	HINATA_NFKB_UP	6	1.53	0.032
Cell death	APOPTOSIS	6	1.55	0.034
	BRENTANI_DEATH	6	1.55	0.034
	APOPTOSIS_GENMAPP	5	1.56	0.035
Cell cycle	CELL_CYCLE_KEGG	6	1.52	0.044
	CELL_CYCLE	6	1.52	0.044
	TAKEDA_NUP8_HOXA9_16D_DN	11	1.43	0.045

**Function/Pathway**	**Gene sets enriched in resistant patients**	**Size**	**NES**	**NOM p-val**

T lymphocyte proliferation	GOLDRATH_HP	14	1.61	0.008
EGR2-regulated genes	LE_MYELIN_UP	6	1.58	0.009
Genes involved in cholera infection	HSA05110_CHOLERA_INFECTION	5	1.63	0.023
Myeloma cell growth and survival	CHAUHAN_2ME2	5	1.50	0.042
DNA and RNA metabolism	WERNER_FIBRO_DN	5	1.45	0.059
p53 response, anti-apoptosis, DNA repair	UVB_SCC_DN	6	1.39	0.086
Myc-regulated genes	MYC_ONCOGENIC_SIGNATURE	8	1.37	0.061
Functions of mature T lymphocytes	LEE_TCELLS2_UP	46	1.27	0.084
Hematopoeitic stem cell genes	BYSTRYKH_HSC_TRANS_GLOCUS	29	1.38	0.070
APC-regulated genes	SANSOM_APC_LOSS5_UP	10	1.47	0.084

NES Normalized Enrichment Score, NOM p-val Nominal p-value

A related analysis identified 316 and 352 genes differentially regulated after two and nine days of treatment, respectively, representing biological functions very similar to those regulated after one day of treatment (data not shown).

### Cytotoxicity of fludarabine on CLL samples in vitro

As a first step to validate *in vitro *some of the candidate marker genes identified above, we carried out cytotoxicity assays on cells from a cohort of CLL patients (N = 21) with the aim to identify fludarabine-sensitive and -resistant cells. The definition of resistance to fludarabine *in vitro *is still a matter of debate; figures have been proposed that range from 20% of cell death following a 24-h exposure to 1 μM fludarabine [21] to an IC_50 _of 10 μM after 72h of treatment [22], and no consensus exists about the dose and time of treatment for *in vitro *studies. In our experiments, cytotoxicity was measured 48h after exposure to fludarabine (0-10 μM) and resistance was defined using a threshold cut-off based on the response obtained with all 21 samples. Thus, although we observed a wide distribution of responses to the treatment among patients, two groups could be clearly identified, and patients displaying an IC_50 _> 7 μM after 48h of treatment *in vitro *were considered resistant (Additional file 7). Sixteen over twenty-one (76.2%) CLL patients were identified as sensitive to fludarabine *in vitro *as the inhibitory concentration (IC_50_) was lower or equal to 4 μM, which is very close to maximum dose achievable in clinics (4.5 μM, [14]). Five patients were classified as resistant (IC_50 _> 7 μM), of which four had an IC_50 _greater than 10 μM.

We then used these two groups of cells to assess the expression of our candidate marker genes and miRNAs (see below) *in vitro*.

### Validation of gene and miRNA markers of cell resistance to fludarabine

**Figure 4:**
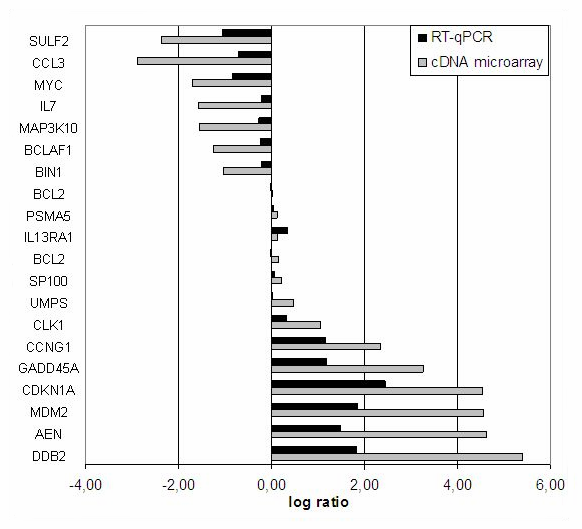
**Validation of cDNA microarray gene expression data by RT-qPCR**. Twenty differentially regulated genes identified in the microarray analysis were validated in the four CLL cases sensitive to fludarabine by RT-qPCR. B lymphocytes were isolated from blood of patients treated 24h *in vivo *with fludarabine. Expression levels of the targets were normalized to both housekeeping genes and Ct values obtained before treatment were used as calibrators. See Methods for details.

**Figure 5:**
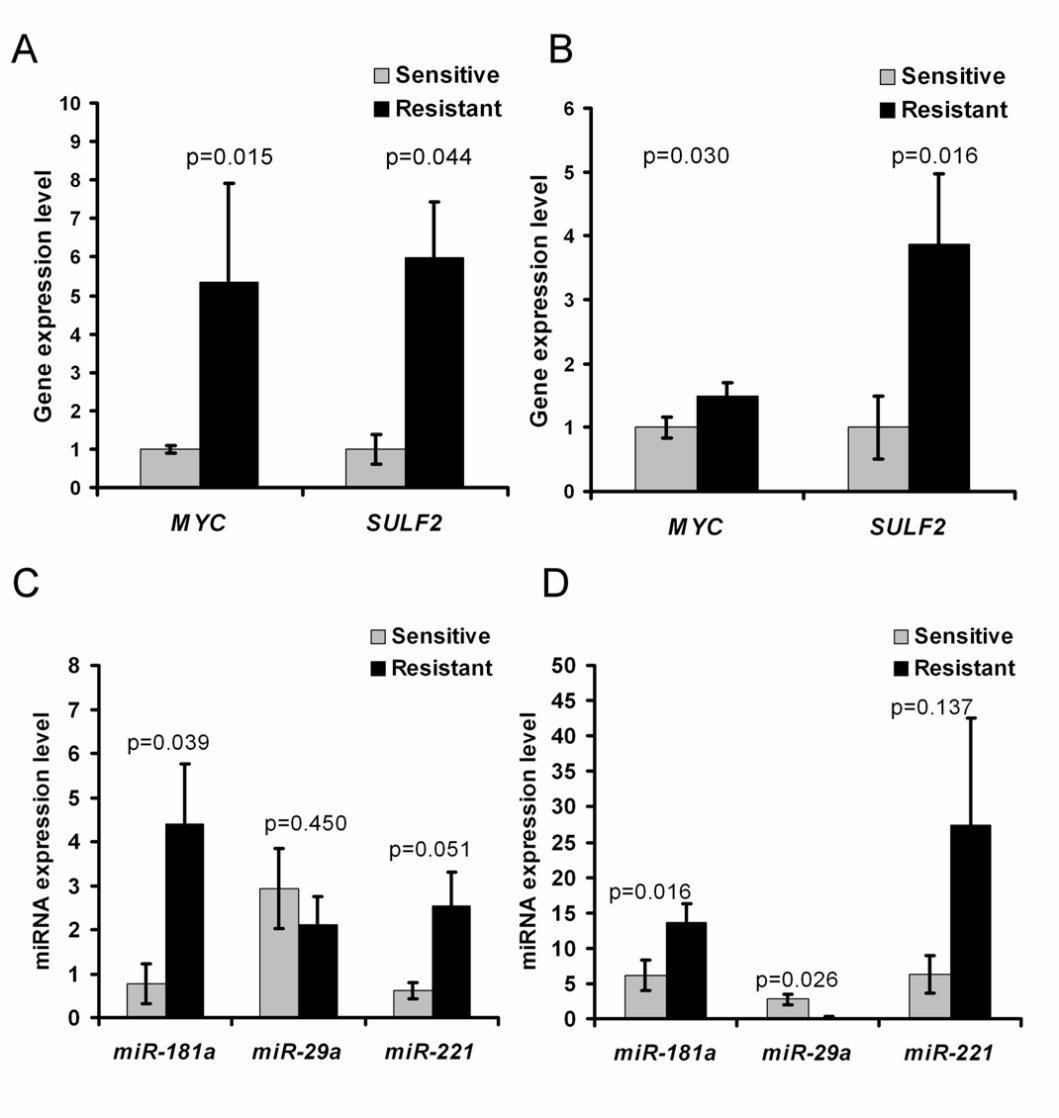
**Relationship between gene expression before treatment and sensitivity to fludarabine in CLL patients**. Expression levels of the targets were investigated by RT-qPCR and normalized to both housekeeping genes. The average expression level in sensitive patients was used as calibrator. Expression levels presented as mean values ±SEM were investigated on two groups of CLL patients; eighteen patients *in vivo *(8S and 10R) and twenty-one patients *in vitro *(16S and 5R). Statistical analysis was performed by 2-sided t-test and levels were considered to be significantly different when p values were lower than 0.05. ***A and B***. The expression of *MYC *and *SULF2 *was measured in CLL B cells of patients before their treatment with fludarabine *in vivo *(A) and *in vitro *(B). ***C and D***. The expression of miR-29a, miR-181a, and miR-221 was investigated in CLL B cells of patients before their treatment with fludarabine *in vivo *(C) and *in vitro *(D).

Microarray data were first validated by RT-qPCR in samples CLL-1 to -6. Twenty genes with various intensities of expression are presented in Fig. 4. Although some discrepancies of intensity were observed, overall gene expression profiles assessed by qRT-PCR after 24h of treatment with fludarabine were consistent with cDNA microarray data. Interestingly, when qPCR were performed to validate microarray data, we observed that the baseline expression of several genes greatly differed between CLL patients before treatment. Consistent with our previous observations, lower levels of transcripts of genes involved in early DNA damage detection and *p53 *signaling were observed in resistant compared to sensitive patients (Table 3). Transcripts of other genes implicated in DNA repair and proliferation were in contrast more abundant. Interestingly, *MYC *and *SULF2 *transcripts were identified as strongly over-represented in B cells of patients CLL-3R and -6R resistant to fludarabine *in vivo *(20-34 and 72-157 fold, respectively). This observation was confirmed on a larger cohort of CLL patients treated with fludarabine *in vivo *(N = 18, Fig. 5A) or *in vitro *(N = 21, Fig. 5B) with both genes being significantly over-expressed in samples of resistant patients obtained before treatment (p < 0.05).

**Table 3:** Transcript levels of prognosis gene markers in B cells of CLL patients before treatment with fludarabine.

	**CLL-1**^1^	CLL-2	**CLL-3R**^2^	CLL-4	CLL-5	**CLL-6R**^2^
***ATM***	1.00 ^3^	1.10	0.42 ^3^	1.91	0.52 ^3^	2.32
***BLM***	1.00	0.54	1.32	0.79	0.92	2.06
***CDK2AP1***	1.00	0.52	2.59	0.33	0.70	1.75
***DCLRE1A***	1.00	1.26	2.84	0.12	1.27	3.11
***MYC***	1.00	1.36	20.33 ^4^	0.95	1.42	34.39 ^4^
***PCNA***	1.00	0.43	2.85	0.59	0.61	1.49
***SULF2***	1.00	0.4	72.6	4.8	2.5	157.4
***TP53***	1.00	0.94	0.04 ^5^	1.12	0.70	0.62 ^5^
***UMPS***	1.00	0.73	1.33	0.71	0.73	1.53
***XPC***	1.00	0.98	0.23	1.43	1.56	0.43

^1 ^Ct values from this patient were used for normalization to compare expression levels between patients with the ΔΔCt calculation method.

^2 ^Patient number followed by "R" indicates the refractoriness character to fludarabine.

^3 ^A mono-allelic deletion on 11q22 was observed.

^4 ^A gain on 8q was observed.

^5 ^A mono-allelic deletion on 17p13 was observed.

Three miRNAs, miR-29a, miR-181a, and miR-221, have been shown to be regulated in CLL [8] and play important regulatory roles in oncogenesis and cell cycle regulation [23,24]. In addition, miR-29a and miR-181 are transcriptional targets of *MYC *family members which repress miR-29a [25] but induce miR-181a transcription [26]. This prompted us to also investigate miRNA expression in fludarabine-sensitive and resistant cells. We found that miR-181a and miR-221 were strongly over-represented in cells of resistant patients (p = 0.015 and p = 0.05), and that miR-29a was significantly down-regulated in patients resistant *in vitro *(p = 0.026) (Fig. 5). Other miRNAs (miR-34a, miR-150*, and let-7e) were also repressed but not significantly (p > 0.05, data not shown).

In summary, we showed in cells of CLL patients resistant to fludarabine an over-expression of *MYC*, *SULF2*, miR-181a, and miR-221 together with a decrease in miR-29a level.

## Discussion

The present study was first designed to improve our understanding of fludarabine-mediated killing of CLL cells *in vivo*. The response of cellular emergency mechanisms to DNA damage includes the transcriptional activation of mediators regulating cell cycle, DNA repair or apoptosis. Here we observed in sensitive B cells a strong up-regulation of p53-target genes (Fig. 1A and Additional file 4); thus confirming the results of previous *in vivo *studies [15,20]. We also detected a marked increase in the level of *CDK4, PLK2*, and cyclin G1 and a down-regulation of the cdk-inhibitor *p27*, suggesting regulatory roles of these genes after fludarabine treatment in cells undergoing apoptosis. Novel regulators identified in this study (Additional file 4), such as the p53-target exonuclease *AEN*, the ER-localized scotin *SHISA5*, the speckle-type POZ protein *SPOP*, the Polo-like kinase *PLK3 *and the apoptosome inducer *ANP32A*, are required for DNA degradation and ER stress during p53-dependent cell cycle arrest and apoptosis [27-31]. Finally, *CASP2*, which is involved in p53-mediated apoptosis [32], and its inducer *ITGB3BP *were up-regulated too, further supporting the role of caspase-2 in the mechanisms underlying the cytotoxicity of fludarabine [32].

Albeit delayed, partial cellular death and induction of apoptosis-related genes were observed in the monoclonal population of resistant patients. This suggests that fludarabine entered the cells and was efficiently activated to its phosphorylated form. Therefore we did not investigate the biochemical aspects of resistance but we focused on the molecular response of the resistant cells. While fludarabine is known to inhibit DNA repair, we observed an over-expression of genes related to cell cycle, growth and survival, and DNA and RNA metabolism suggesting that cellular maintenance mechanisms operate in lymphocytes from resistant patients (Table 2 and Additional file 5). This combination of factors could explain resistance as cells exhibit a reduced p53-response whilst displaying active DNA repair and proliferative responses. Factors negatively regulating p53 activation and increasing its degradation were also over-expressed (*DDX17*, *SNCB*, *SOD1*, and *TRIM28 *[*KAP1*]). Interestingly, *KAP1 *protects cells from apoptosis in response to DNA damage [33] and represses *p21*, *GADD45A*, *Puma*, *Noxa*, and *Bax *transcription, suggesting the repression of p53-dependent apoptotic effectors by *KAP1*-dependent mechanisms [34].

Recently, the availability of growth factors and angiogenesis have been recognized as important factors in CLL pathology [35] Our analysis of the pathways regulating cellular growth and proliferation revealed that transcripts of the 6-O endosulfatase *SULF2*, a novel p53-target gene [36], were differentially regulated in patients and dramatically more abundant in cells of resistant patients (Fig. 5). Contradictory functions of *SULF2 *have been reported in different models. Sulf2 has been shown to promote cell proliferation and migration by releasing VEGF and SDF-1 from heparin/heparin sulfate proteoglycans in the extracellular matrix [37]. The abundance of *SULF2 *transcripts, observed in this study in resistant patients prior to treatment, is in agreement with its proposed pro-angiogenic and oncogenic effects [38-40]. However fludarabine was found to increase the level of *SULF2 *mRNA in cells of sensitive patients only (Additional file 4). This finding also supports the notion that *SULF2 *is induced by anti-metabolites [41] and can be a potent inhibitor of hematologic tumor growth *in vivo *[42]. We also detected a fludarabine-induced over-expression of the growth inhibitors *SPRY1 *and *CAV1 *[43] mRNA in all conditions. Further investigations about tumor microenvironment will be required to understand the growth of malignant CLL cells *in vivo*.

Next we sought to investigate the genomic abnormalities associated with resistance. In addition to gains on chr7 (*MDR *genes) previously described [44] and a 9p deletion targeting *SMARCA2 *necessary for induction of p53 targets [45], we detected an important gain on the 15q arm. The presence of p53-cooperating genes on 15q reinforces the interest for this aberration in atypical CLL treated with DNA damage-inducing agents. In addition to these aberrations, we identified three isochromosomes in CLL cells leading to loss of heterozygosity. Thus isochromosomes may have clinical implications even though they are probably not primary lesions. As a consequence of the presence of the idic(8q), we observed a gain on 8q bearing the c-*MYC *oncogene in some resistant patients, which led us to investigate *MYC *expression in our samples. We observed the strong over-expression of *MYC *transcripts in CLL cells of resistant patients before treatment (Fig. 5A). This regulation was later confirmed in cells resistant to fludarabine *in vitro*, which also displayed higher levels of *SULF2 *(Fig. 5B).

We then considered Myc transcriptional targets and identified several differentially expressed genes and miRNAs that are direct targets of Myc in CLL B cells. An important conclusion of our work is that the Myc target gene network was up-regulated in cells that were resistant to fludarabine, whereas the p53 target gene network was down-regulated in these cells. For instance, over-expression of the Myc targets *CDK4 *and *CCNB1 *(cyclinB1)[46,47], which is suggestive of cell cycle progression, was observed in cells from our resistant patients. Interestingly, c-Myc and p53 have opposite effects on the regulation of cyclin B1, and induction of chromosomal instability by cyclin B1 over-expression was recently reported [47]. Likewise, Myc represses p53-induced apoptosis in leukemia cells and selectively prevents p53 target gene transcriptional activation [48,49]. Differentially expressed Myc targets also included miRNAs. Thus, miR-29a and miR-181a are both confirmed transcriptional targets of *MYC *family members which repress miR-29a [25] but induce miR-181a transcription [26]. Interestingly, miR-181a and miR-221 were strongly over-expressed in the cells of resistant patients (Fig. 5 C and D), confirming a previous association of miR-181a and miR-221 with poor prognosis [8] but contradicting another report that low miR-181a expression levels are associated with disease progression [9]. High levels of miR-181a and miR-221 also point to cell cycle progression as both miRNAs repress *CDKN1B *(p27) expression in hematologic diseases [23,50] and p27 was also found down-regulated in resistant cells (Additional file 5). Notably, miR-181a also targets phosphatases that modulate the activity of signaling kinases such as Zap-70 [51]; thus reinforcing the interest for this miRNA in CLL research. We also noticed that miR-29a was down-regulated in cells of resistant patients. In CLL, miR-29a levels have been reported to inversely correlate with *TCL1 *oncogene expression [24]. Studies using other cellular models indicate that miR-29a can activate p53 and induce apoptosis in a p53-dependent manner [52]. Our observation that the levels of miR-29a are lower in resistant patients is in agreement with these previous findings and suggests the importance of this regulation in aggressive CLL cases. Taken together, our data therefore underline the crucial and opposite role of the Myc and p53 gene regulatory networks in determining the cell response to fludarabine in CLL. Furthermore they underline the attraction of using Myc inhibition as an approach for improving CLL therapy. Amplification of Myc (i(8q)) in some CLL cases [53] reinforces this notion [54].

## Conclusions

Although a recent report reinforces the prominent role of *TP53 *mutation as a prognosis tool for CLL [55], our work lends supports to other studies which have challenged the prognostic value of aberrations in master regulators such as *ATM *and *TP53 *[56,57]. Furthermore, our research considerably extends previous studies by identifying the oncogene *MYC *as over-expressed in B cells of CLL patients resistant to fludarabine *in vivo *and *in vitro*, and establishing the aberrant regulation of *Myc*-transcriptional target genes and miRNAs in these patients. Our data therefore support the importance of *MYC *in CLL pathology and its association with poor prognosis, as suggested by cytogenetic analyses [58]. They also underline the importance of the tumor microenvironment in the progression of CLL *in vivo*. Furthermore our work leads us to propose that the expression levels of *MYC, SULF2, miR-29a, miR-181a *and *miR-221a *measured in CLL patients could be of interest during clinical trials and may help to predict the chemo-refractory character of patients. Finally, Myc inhibition may be of high value in the treatment of poor prognosis CLL patients.

## Competing interests

The authors declare that they have no competing interests.

## Authors' contributions

EM designed the study, performed research, analyzed data and wrote the paper. VP, LV, and EVD designed the study and wrote the paper. HAP performed cytogenetic analysis. TW performed sequencing and data analysis. VEK, NA, KVM, BL, and FB performed research and analyzed data. AM performed data mining analyses. PCL, AD, CD, FR and GB recruited patients, obtained written consents, and collected clinical data. All authors have read and approved the final manuscript.

## Supplementary Material

Additional file 1 **Clinical characteristics of B-CLL patients**. Data concerning CLL patients treated *in vivo *and used in the study were gathered at sample collection (gender, age, stage, previous treatments and lymphocyte counts), obtained after blood processing and analysis (cytogenetics) or during the clinical follow-up of the patients (time to progression). Genomic abnormalities were detected by CGH array and FISH in B cells of CLL patients. Click here for file

Additional file 2 **Accumulation of genomic abnormalities in CLL B cells resistant to fludarabine**. (A) FISH analysis confirmed the 15q22 and 17q21 gains without any fusion. Green signal: RARα locus; red signal: PML locus. (B) Monoallelic 17p13 deletion (p53) due to isochromosome 17 in 92% of cells and chromosome 12 trisomy together with p53 deletion present in 7.5% of cells. Green signal: 12cen; red signal: TP53 locus. (C) Monoallelic 13q14 deletion in 90% of cells. Green signal: 13 qter; red signal: 13q14 region. (D) Presence of IGH-BCL1 fusion confirming the t(11;14). Green signal: IGH locus; red signal: BCL1 locus. Nuclei were counterstained with 4,6-diamino-2-phenylindole (DAPI). Pictures are representative of CLL-3R and -6R resistant patients. Click here for file

Additional file 3 **Profiles of chromosomes 3, 8, 15, and 17 obtained by CGH-array analysis**. Chromosome profiles were obtained after hybridization of blood B cells DNA. Similar aberrations were observed on blood B cells from resistant patients. (A) The graphical view (CGH analytics, Agilent) of isochromosome idic(3)(p12) was obtained from patient CLL-3R. (B-D) The idic(8)(p12), the gain on chromosome 15, and the idic(17)(p12) were obtained from patient CLL-6R. Click here for file

Additional file 4 **Level of selected differentially expressed genes in CLL B cells of sensitive patients following treatment with fludarabine *in vivo***. Gene expression profiling of B cells from CLL patients sensitive to fludarabine indicated the regulation of genes involved in the regulation of cell death, cell cycle and in the response to DNA damage. Fold change of selected genes are presented at different time points. Click here for file

Additional file 5 **Level of selected differentially expressed genes in CLL B cells of resistant patients following treatment with fludarabine *in vivo***. Gene expression profiling of B cells from CLL patients resistant to fludarabine indicated the regulation of genes involved in the regulation of DNA repair, cell growth and proliferation and cell death. Fold change of selected genes are presented at different time points. Click here for file

Additional file 6 **SAM analysis of regulated genes in B cells of resistant and sensitive CLL patients**. Genes up-regulated in cells of resistant and sensitive CLL patients were identified after 24h of treatment with fludarabine *in vivo*. The gene expression profiles from both groups were directly compared by a SAM 2-class analysis (unpaired, 100 permutations). The false discovery rate (FDR) was set at 5% and only significant genes were exported. The score *d *denotes the standardized change in expression. Click here for file

Additional file 7 **Cytotoxic effect of fludarabine on CLL cells *in vitro***. The cytotoxicity of fludarabine (0-10 μM) was examined on cells of CLL patients (N = 21) after 48h of treatment. Patients exhibiting an IC_50 _greater than 7 μM after 48h of treatment with fludarabine were considered to be resistant (N = 5). When the IC_50 _was greater than 10 μM, the value was set at 10 μM. Click here for file
