# Supplementary material for: Determination of genes and microRNAs involved in the resistance to fludarabine in vivo in chronic lymphocytic leukemia
Source: Mol Cancer. 2010 May 20;9:115. doi: 10.1186/1476-4598-9-115 (PMC2881880; doi:10.1186/1476-4598-9-115)
Supplement: Additional file 1 — Clinical characteristics of B-CLL patients. Data concerning CLL patients treated in vivo and used in the study were gathered at sample collection (gender, age, stage, previous treatments and lymphocyte counts), obtained after blood processing and analysis (cytogenetics) or during the clinical follow-up of the patients (time to progression). Genomic abnormalities were detected by CGH array and FISH in B cells of CLL patients. [file 1476-4598-9-115-S1.PDF]

**Additional file 1. Clinical characteristics of B-CLL patients**

| Patient | Sex/ age | Rai stage | IgVH status | Losses | Gains | *TP53* status | Lympho count before F | Lympho count after F | F cycles | TTP | Previous treatments |
|---|---|---|---|---|---|---|---|---|---|---|---|
| **CLL-1S** | M/77 | IV | VH1-46<br>UM | 1q32.1<br>11(q14q23)<br>13q14 | | Wt | 124,000 | 4,800 | 3 | NR | Ch<br>FCh |
| **CLL-2S** | F/69 | III | VH1-18<br>M | 13q14 | | Wt | 80,000 | 1,300 | 1 | 6 | None |
| **CLL-3R** | M/76 | IV | VH4-61<br>M | 3p<br>8p<br>9(p24q21)<br>11(q22q25)<br>12(p13p12)<br>13q14<br>14(q24q24)<br>14(q32q32)<br>15q15q26<br>17p | 3q<br>8q<br>17q | Del/Mut<br>Codon 239<br>Δ2nt=>stop | 220,000 | 50,000 | 1 | 0 | Ch<br>F<br>FChRx |
| **CLL-4S** | F/63 | IV | VH3-30<br>UM | 4(q33q35)<br>13q14 | 3(q26q29)<br>21(q21q22) | Wt | 57,000 | 200 | 3 | NR | Ch<br>Rx |
| **CLL-5S** | M/71 | I | VH3-74<br>M | 4(p16q22)<br>11(q22q24)<br>13q12<br>13q14<br>17p13<br>18(p11q32) | | V274G | 102,000 | 3,100 | 1 | NR | Ch |
| **CLL-6R** | F/79 | III | VH2-70<br>M | 8p<br>17p<br>7q22<br>7(q21q21)<br>7(q31q31)<br>13q14<br>15(q15q26) | 8q<br>17q<br>3(q12q29)<br>7(p22q11) | Del/Wt | 74,000 | 48,600 | 2 | 4 | None |

| | | | | | | | | | | | |
|---|---|---|---|---|---|---|---|---|---|---|---|
| **CLL-7R** | M/57 | I | NA | t(1;4)(q11;q35)<br>t(1;6)(q11;p11)<br>t(1;12)(q11;p11) | | Wt | 41,400 | 5,600 | 2 | 2 | Ch |
| **CLL-8S** | M/57 | II | NA | NA | | Wt | 67,900 | 900 | 6 | 23 | Ch<br>Mini-CHOP<br>x 4 |
| **CLL-9R** | M/43 | 0 | VH3-33<br>UM | NA | | Wt | 129,000 | 1,800 | 6 | 6 | None |
| **CLL-10S** | M/72 | 0 | VH3-33<br>UM | | +12 | Wt | 228,000 | 1,000 | 6 | NA | None |
| **CLL-11R** | M/74 | 0 | NA | NA | | Wt | 430,000 | 20,000 | 3 | 3 | None |
| **CLL-12R** | M/66 | II | NA | | +12 | Wt | 115,000 | 2,000 | 6 | NA | None |
| **CLL-13R** | M/64 | I | VH1-69<br>UM | 11q22 | | Del | 89,000 | 4,000 | 3 | 0 | CHOP x 3 +<br>autologous<br>transplant |
| **CLL-14R** | M/64 | I | VH3-48<br>UM | 11q22<br>13q14 | | Del | 330,000 | 50,000 | 2 | 0 | None |
| **CLL-15S** | M/60 | I | NA | 13q14 | | Wt | 138,000 | 2,700 | 6 | NA | None |
| **CLL-16R** | M/75 | II | NA | | +12 | NA | 30,700 | 1,000 | 6 | 10 | Ch<br>Mini-CHOP<br>x 12 |
| **CLL-17R** | F/63 | IV | VH3-13<br>M | 13q14 | | Wt | 144,000 | 2,500 | 6 | NA | None |
| **CLL-18S** | M/57 | II | NA | 13q14 | | Wt | 11,700 | 800 | 6 | 22 | None |

**<u>Notes</u>:** Ch indicates Chlorambucil; CHOP, cyclophosphamide, doxorubicin, vincristine, and prednisolone; F, Fludarabine; M, Mutated; NA, Not available; ND Not determined; NR, Not reached; UM, Unmutated; R, Resistant to fludarabine; Rx, Rituximab; TTP, Time to progression (in months).
