# Supplementary material for: Determination of genes and microRNAs involved in the resistance to fludarabine in vivo in chronic lymphocytic leukemia
Source: Mol Cancer. 2010 May 20;9:115. doi: 10.1186/1476-4598-9-115 (PMC2881880; doi:10.1186/1476-4598-9-115)

Additional file 2. Accumulation of genomic abnormalities in CLL B cells resistant to fludarabine.

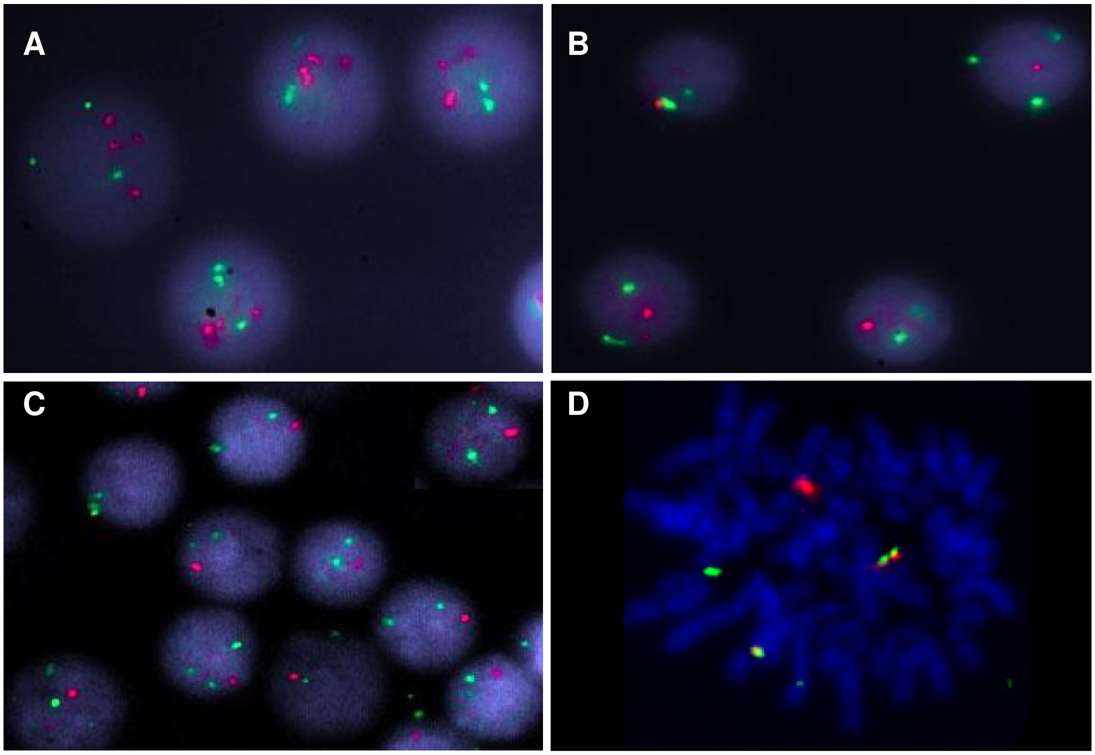

Supplement: Additional file 2 — Accumulation of genomic abnormalities in CLL B cells resistant to fludarabine. (A) FISH analysis confirmed the 15q22 and 17q21 gains without any fusion. Green signal: RARα locus; red signal: PML locus. (B) Monoallelic 17p13 deletion (p53) due to isochromosome 17 in 92% of cells and chromosome 12 trisomy together with p53 deletion present in 7.5% of cells. Green signal: 12cen; red signal: TP53 locus. (C) Monoallelic 13q14 deletion in 90% of cells. Green signal: 13 qter; red signal: 13q14 region. (D) Presence of IGH-BCL1 fusion confirming the t(11;14). Green signal: IGH locus; red signal: BCL1 locus. Nuclei were counterstained with 4,6-diamino-2-phenylindole (DAPI). Pictures are representative of CLL-3R and -6R resistant patients. [file 1476-4598-9-115-S2.PDF]
