# Supplementary figures and images for: Determination of genes and microRNAs involved in the resistance to fludarabine in vivo in chronic lymphocytic leukemia
Source: Mol Cancer. 2010 May 20;9:115. doi: 10.1186/1476-4598-9-115 (PMC2881880; doi:10.1186/1476-4598-9-115)

**Additional file 3. Profiles of chromosomes 3, 8, 15, and 17 obtained by CGH-array analysis.**

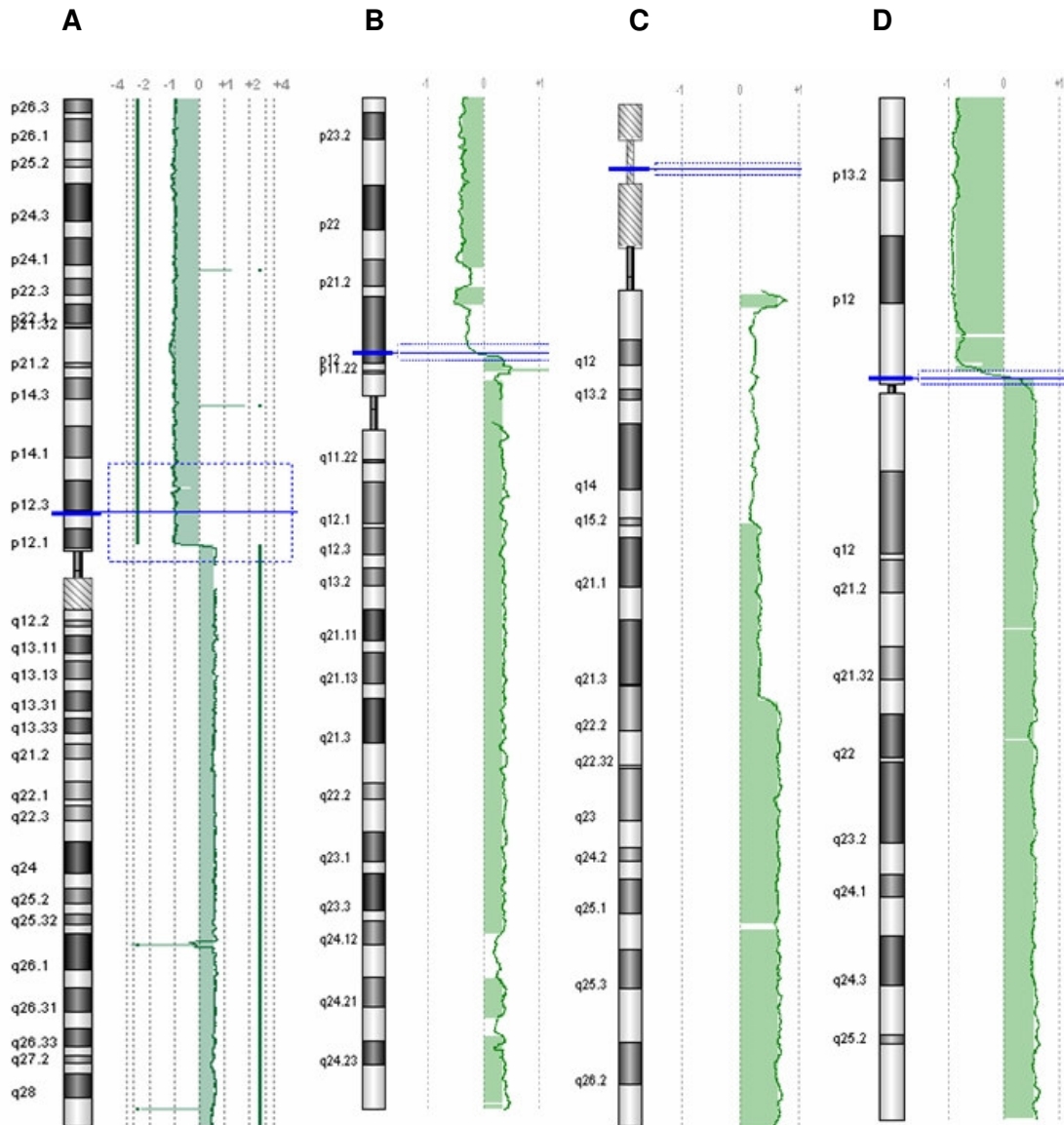

Supplement: Additional file 3 — Profiles of chromosomes 3, 8, 15, and 17 obtained by CGH-array analysis. Chromosome profiles were obtained after hybridization of blood B cells DNA. Similar aberrations were observed on blood B cells from resistant patients. (A) The graphical view (CGH analytics, Agilent) of isochromosome idic(3)(p12) was obtained from patient CLL-3R. (B-D) The idic(8)(p12), the gain on chromosome 15, and the idic(17)(p12) were obtained from patient CLL-6R. [file 1476-4598-9-115-S3.PDF]

Additional file 7. Cytotoxic effect of fludarabine on CLL cells *in vitro*.

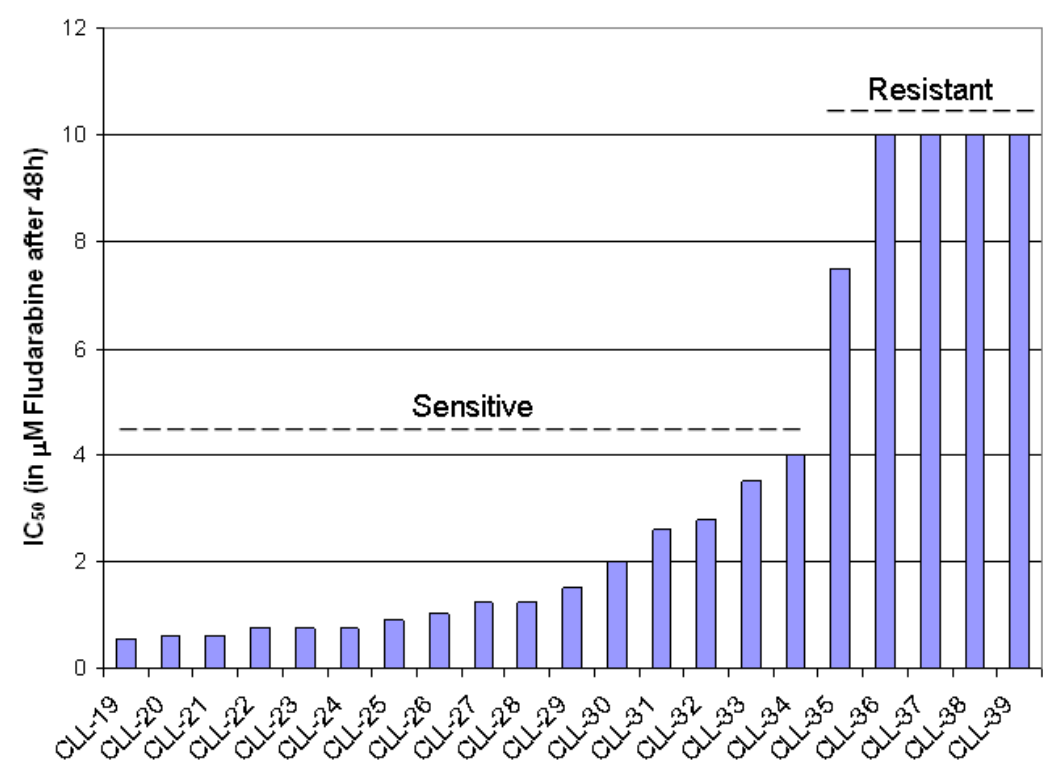

Supplement: Additional file 7 — Cytotoxic effect of fludarabine on CLL cells in vitro. The cytotoxicity of fludarabine (0-10 μM) was examined on cells of CLL patients (N = 21) after 48h of treatment. Patients exhibiting an IC50 greater than 7 μM after 48h of treatment with fludarabine were considered to be resistant (N = 5). When the IC50 was greater than 10 μM, the value was set at 10 μM. [file 1476-4598-9-115-S7.PDF]
