# Supplementary material for: Determination of genes and microRNAs involved in the resistance to fludarabine in vivo in chronic lymphocytic leukemia
Source: Mol Cancer. 2010 May 20;9:115. doi: 10.1186/1476-4598-9-115 (PMC2881880; doi:10.1186/1476-4598-9-115)
Supplement: Additional file 4 — Level of selected differentially expressed genes in CLL B cells of sensitive patients following treatment with fludarabine in vivo. Gene expression profiling of B cells from CLL patients sensitive to fludarabine indicated the regulation of genes involved in the regulation of cell death, cell cycle and in the response to DNA damage. Fold change of selected genes are presented at different time points. [file 1476-4598-9-115-S4.PDF]

Additional file 4. Relative expression levels of selected differentially regulated genes in CLL B cells of sensitive patients following treatment with fludarabine *in vivo*

Biological functions		p53 target*	Fold change		
Gene name	Description		T0-T1	T1-T2	T2-T9
**Response to DNA damage / DNA repair**					
DDB2	DNA damage-binding protein 2	x	42.52	6.19	0.65
MDM2	Ubiquitin-protein ligase E3	x	23.59	11.79	0.25
PCNA	Proliferating cell nuclear antigen	x	12.64	3.05	0.50
XPC	DNA-repair protein complementing XP-C cells	x	7.06	2.97	0.46
POLM	DNA polymerase mu		1.95	— †	2.19
BTG2	NGF-inducible anti-proliferative protein	x	1.93	1.45	2.14
RAD51	DNA repair protein RAD51 homolog 1		1.77	1.95	—
RRM2B	ribonucleotide reductase M2 B (p53R2)	x	1.75	1.75	—
XRCC1	X-ray repair cross-complementing protein 1		1.64	1.60	2.00
CIZ1	Cip1-interacting zinc finger protein		1.60	1.91	—
CETN2	Centrin-2		0.69	0.65	—
BRCA2	Breast cancer type 2 susceptibility		0.65	—	0.66
RAD21	Double-strand-break repair protein		0.60	—	0.62
RAD23B	UV excision repair protein RAD23 homolog B		0.58	0.62	—
XPA	DNA-repair protein complementing XP-A cells		0.51	0.18	—
POLS	DNA polymerase sigma		—	1.82	2.25
**DNA replication**					
MYST2	Histone acetyltransferase 2		1.88	1.85	—
MYST3	Histone acetyltransferase 3		1.87	1.88	—
RAD51L3	DNA repair protein RAD51 homolog 4		0.59	0.65	—
CHRAC1	Chromatin accessibility complex 1		0.47	0.63	—
CDK2AP1	Cyclin-dependent kinase 2-associated protein 1		0.46	0.41	0.66
**Cell stress response**					
GADD45A	Growth arrest and DNA-damage-inducible, alpha	x	9.58	2.77	0.44
IER5	Immediate early response gene 5	x	5.13	2.30	—
DDIT4	DNA-damage-inducible transcript 4	x	2.53	—	2.83
TP53AP1	TP53 activated protein 1	x	2.51	2.04	0.70
DDIT3	DNA damage-inducible transcript 3		—	6.06	—
**Programmed cell death**					
AEN	Apoptosis enhancing nuclease (ISG20L1)	x	27.47	2.50	0.21
BAX	BCL2-associated X protein	x	7.52	3.34	—
TNFRSF10B	TNF receptor superfamily, member 10b (DR5)	x	6.36	4.89	—
YAP1	65 kDa Yes-associated protein	x	4.89	4.38	2.57
ZMAT3	p53 target zinc finger protein (Wig1)	x	4.79	2.89	—
ANP32A	Acidic leucine-rich nuclear phosphoprotein 32 A		4.47	4.20	3.12
ITGB3BP	Beta3-endonexin		3.73	6.73	3.56
BBC3	BCL2 binding component 3 (Puma)	x	2.33	2.35	—
DAXX	Death domain-associated protein 6		2.33	2.57	—
PERP	p53 apoptosis effector	x	2.27	—	—
LITAF	Lipopolysaccharide-induced TNF-alpha factor		2.22	—	2.58
BCL2L11	Bcl-2-like protein 11 (Bim)	x	2.17	1.78	—
MOAP1	Modulator of apoptosis 1		2.14	2.62	—
SPOP	Speckle-type POZ		2.03	1.84	4.47
SHISA5	Shisa homolog 5 *(Scotin)*	x	1.78	2.06	2.14
PIG8	p53-induced protein 8	x	1.77	—	—
P2RX5	P2X purinoceptor 5		1.65	1.45	2.64
RASSF1	Ras association domain-containing protein 1		1.61	2.11	1.30
PMAIP1	PMA-induced protein 1 (Noxa)	x	—	3.73	0.51
**Cell survival**					
TRIAP1	TP53-regulated inhibitor of apoptosis 1	x	14.83	2.43	—

BIRC5	Baculoviral IAP repeat-containing 5 (survivin)		0.85	0.65	—
EEF1A1	Translation elongation factor 1 alpha 1	x	0.37	0.62	0.41
SCD	Stearoyl-CoA desaturase	x	—	0.52	1.11
**Cell cycle / proliferation**					
SULF2	Sulfatase 2	x	31.56	17.03	0.18
CDKN1A	Cyclin-dependent kinase inhibitor 1 (p21)	x	23.43	8.11	0.70
PLK2	Serine/threonine-protein kinase	x	5.35	5.17	0.44
CCNG1	Cyclin-G1	x	5.13	2.64	0.44
RINT1	RAD50 interactor 1		2.51	2.81	2.28
CAV1	Caveolin-1	x	2.11	1.69	—
CCND2	Cyclin-D2		1.91	1.99	—
CDK4	Cell division protein kinase 4		1.85	2.13	2.10
PLK3	Serine/threonine-protein kinase		1.95	2.13	—
HADC1	Histone deacetylase 1		0.63	0.65	0.78
RB1CC1	RB1-inducible coiled-coil protein 1		0.56	0.59	0.50
DUSP2	Dual specificity protein phosphatase 2 (*PAC-1*)	x	0.53	0.14	3.23
IL-7R	Interleukin-7 receptor alpha chain precursor		0.50	0.38	0.55
IL-7	Interleukin-7 precursor		0.45	0.51	0.71
BRCC3	BRCA1/BRCA2-containing complex subunit 3		0.44	0.64	0.84
MYC	Proto-oncogene c-Myc		0.39	0.50	—
MAX	MYC associated factor X		0.32	0.48	—
CDKN1B	Cyclin-dependent kinase inhibitor 1B (p27)		—	0.62	0.72
**Others**					
GREB1 beta	Gene regulated in breast cancer 1		5.82	9.45	4.69
SPRY1	Sprouty homolog 1		5.17	5.06	3.14
SNCB	Beta-synuclein		4.72	7.41	3.14
DDX17	DEAD (Asp-Glu-Ala-Asp) box polypeptide 17		2.93	2.50	—
IRF9	Interferon regulatory factor 9		2.14	2.14	3.53
SPT5	Transcription elongation factor SPT5		1.64	1.77	1.65
DLEU2	Deleted in lymphocytic leukemia 2		0.74	0.51	—
POLDIP3	DNA polymerase delta interacting protein 3		0.52	0.68	—
LY75	Lymphocyte antigen 75 precursor (CD205)		0.39	0.36	0.45
CD69	Early activation antigen		0.33	—	0.68
TNFRSF18	TNF receptor superfamily member 18		0.29	0.42	0.77
AUTS2	Autism susceptibility candidate 2		0.20	0.32	—

* The status of each gene as a p53-transcriptional target gene is based on literature.
† The absence of significant gene regulation is marked by "—".
