# Supplementary material for: Determination of genes and microRNAs involved in the resistance to fludarabine in vivo in chronic lymphocytic leukemia
Source: Mol Cancer. 2010 May 20;9:115. doi: 10.1186/1476-4598-9-115 (PMC2881880; doi:10.1186/1476-4598-9-115)
Supplement: Additional file 5 — Level of selected differentially expressed genes in CLL B cells of resistant patients following treatment with fludarabine in vivo. Gene expression profiling of B cells from CLL patients resistant to fludarabine indicated the regulation of genes involved in the regulation of DNA repair, cell growth and proliferation and cell death. Fold change of selected genes are presented at different time points. [file 1476-4598-9-115-S5.PDF]

Additional file 5. Relative expression levels of selected differentially regulated genes in CLL B cells of resistant patients following treatment with fludarabine *in vivo*

Biological functions		p53 target*	Fold change		
Gene name	Description		T0-T1	T1-T2	T2-T9
**Response to DNA damage / DNA repair**					
UMPS	Uridine 5'-monophosphate synthase		8.57	— †	2.45
MCM7	DNA replication licensing factor		2.66	2.27	—
PARP1	Poly [ADP-ribose] polymerase 1		2.51	1.80	2.48
TRIM28	Tripartite motif-containing 28 (*KAP1*)		2.50	—	1.84
ERH	Enhancer of rudimentary homolog		2.38	2.50	—
SOD1	Superoxide dismutase 1		2.28	1.96	—
TERF2IP	Telomeric repeat-binding factor 2-interacting protein 1		2.08	2.30	—
POLE3	DNA directed polymerase, epsilon 3		1.73	2.16	—
RAD51	DNA repair protein RAD51 homolog 1		1.66	2.93	1.92
ATM	Serine-protein kinase		—	1.97	2.60
TOP1MT	DNA topoisomerase I, mitochondrial		—	1.92	1.96
TOP2B	DNA topoisomerase 2-beta		—	0.49	0.68
NOL8	Nucleolar protein 8		—	0.49	0.64
53BP1	Tumor protein p53 binding protein 1	x	—	—	2.13
**Programmed cell death**					
ANP32A	Acidic leucine-rich nuclear phosphoprotein 32 A		4.03	4.63	3.12
ZFP36L2	Zinc finger protein 36, C3H type-like 2	x	3.16	0.24	—
SPOP	Speckle-type POZ protein		2.83	2.07	2.14
SHISA5	Shisa homolog 5 (*Scotin*)	x	1.79	—	2.00
GSK3A	Glycogen synthase kinase-3 alpha		0.60	—	0.64
DAXX	Death domain-associated protein 6		—	2.75	3.10
MOAP1	Modulator of apoptosis 1		—	2.67	1.84
BAX	BCL2-associated X protein	x	—	2.43	2.28
ZMAT3	p53 target zinc finger protein isoform 1 (*Wig1*)	x	—	2.20	1.79
**Cell survival**					
HSPA8	Heat shock cognate 71 kDa protein	x	3.12	2.06	1.78
DFFA	DNA fragmentation factor subunit alpha		1.82	2.25	2.64
**Cell cycle / proliferation / growth**					
ITGB3BP	Beta3-endonexin		3.78	5.35	4.66
CAV1	Caveolin-1		3.18	3.31	—
CCNH	Cyclin-H		3.07	—	2.43
CDK2AP2	Cyclin-dependent kinase 2-associated protein 2		2.69	—	2.68
CCNB1	G2/mitotic-specific cyclin-B1	x	2.38	—	1.74
PCNA	Proliferating cell nuclear antigen	x	2.13	2.99	—
CDK5RAP1	CDK5 regulatory subunit-associated protein 1		1.80	2.64	—
CDK4	Cell division protein kinase 4		1.65	2.01	2.87
CDKN1B	Cyclin-dependent kinase inhibitor 1B (p27)		0.66	—	0.49
EP300	E1A-associated protein p300		0.56	—	0.60
DUSP2	Dual specificity protein phosphatase 2 (*PAC-1*)	x	0.41	0.43	—
CDK2AP1	Cyclin-dependent kinase 2-associated protein 1		0.30	0.47	0.40
SULF2	Sulfatase 2	x	0.22	0.30	0.57
RASSF1	Ras association domain-containing protein 1		—	2.89	2.60
CDKN2D	Cyclin-dependent kinase 4 inhibitor D ($p19^{INK4d}$)		—	2.16	2.25
CDK9	Cell division protein kinase 9		—	2.11	1.89
CCND2	G2/S-specific cyclin-D2		—	2.04	2.82
**Others**					
SNCB	Beta-synuclein		8.88	9.78	11.71
CNIH4	Cornichon homolog 4		4.38	3.32	4.14
DDX17	DEAD (Asp-Glu-Ala-Asp) box polypeptide 17		3.56	2.99	2.58
HLA-DQA1	Major histocompatibility complex, class II, DQ alpha 1		2.90	—	4.13

* The status of each gene as a p53-transcriptional target gene is based on literature.
† The absence of significant gene regulation is marked by “—”.
